# Supplementary material for: Thermochemistry, Tautomerism, and Thermal Stability of 5,7-Dinitrobenzotriazoles
Source: Int J Mol Sci. 2023 Mar 10;24(6):5330. doi: 10.3390/ijms24065330 (PMC10049112; doi:10.3390/ijms24065330)
Supplement: Supplementary file 1 [file ijms-24-05330-s001.zip › ijms-2166646-supplementary.pdf]

Supporting Information

Thermochemistry, Tautomerism, and Thermal Stability of 5,7-Dinitrobenzotriazoles

Igor N. Melnikov [1], Vitaly G. Kiselev [1,2,3], Igor L. Dalinger [4], Alexey M. Starosotnikov [4], Nikita V. Muravyev [1,*], Alla N. Pivkina [1]

[1] Semenov Federal Research Center for Chemical Physics RAS, 4 Kosygina Str., 119991 Moscow, Russia
[2] Novosibirsk State University, 1 Pirogova Str., 630090 Novosibirsk, Russia
[3] Institute of Chemical Kinetics and Combustion SB RAS, 3 Institutskaya Str., 630090 Novosibirsk, Russia
[4] Zelinsky Institute of Organic Chemistry RAS, 47 Leninsky Ave., 119991 Moscow, Russia

Table of contents

S1. The dependences of the heat release and residual mass against heating rate 2
S2. The model-fitting kinetic analysis 3
S3. Gas-phase primary decomposition pathways of DBT and ADBT: high-level quantum chemical calculations 4
S4. Primary decomposition channels for 1H-4,6 and 2H-4,6-tautomers 6
S5. The decomposition mechanism in solution based on PCM calculations 8
S6. Kinetic parameters of the initial decomposition pathways of DBT and ADBT 10
S7. Experimental thermochemical data of DBT 11
S8. Raw quantum chemical data: optimized geometries, electronic energies, and thermal corrections to thermodynamic potentials of all compounds under study ... 12

S1. The dependences of the heat release and residual mass against heating rate

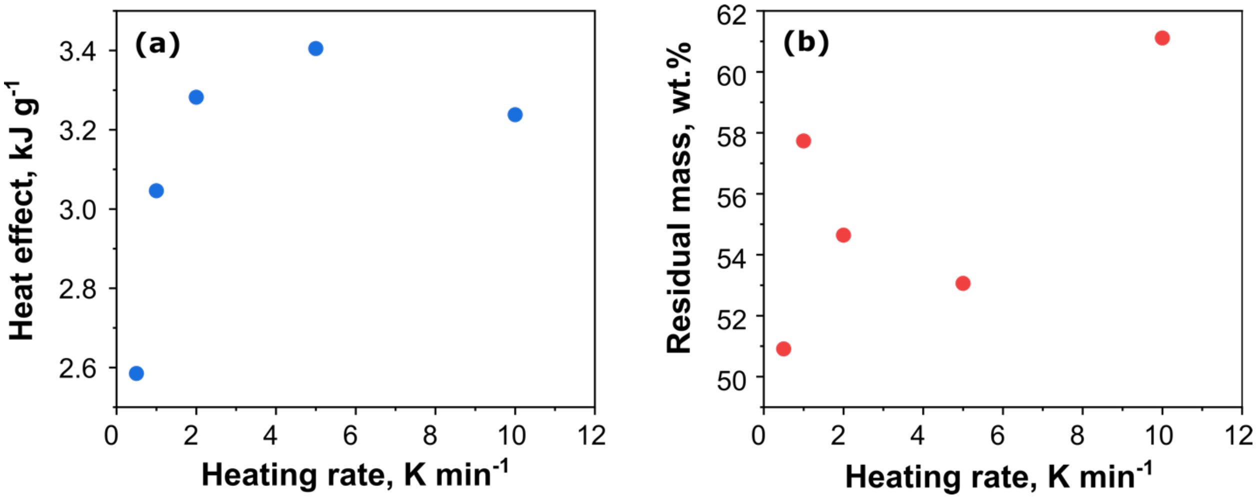

Figure S1. The dependences of the heat effect registered in DSC measurements, (**a**) and residual sample mass, after the DSC tests, (**b**) against heating rates for **DBT**.

S2. The model-fitting kinetic analysis

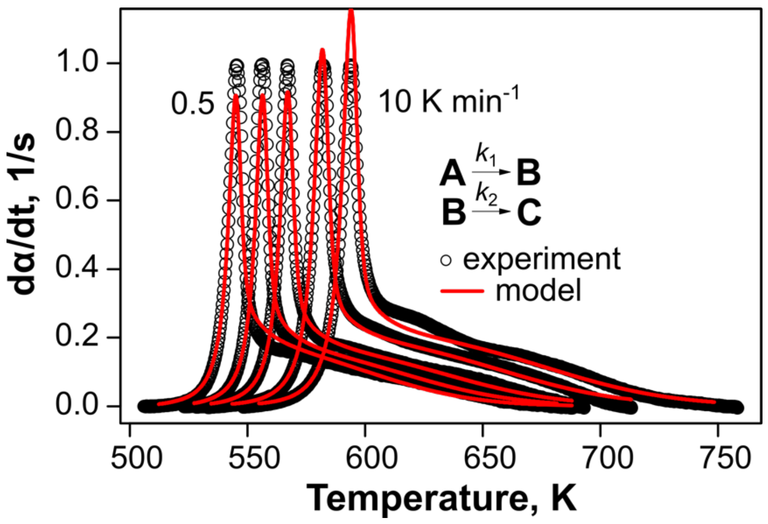

Figure S2. Normalized DSC data for **DBT** (rarefied points) fitted by the kinetic scheme with two consecutive reactions according to ePT equation (red curves). From left to right, the datapoints correspond to 0.5, 1, 2, 5, and 10 K min^{-1} heating rates.

S3. Gas-phase primary decomposition pathways of DBT and ADBT: high-level quantum chemical calculations

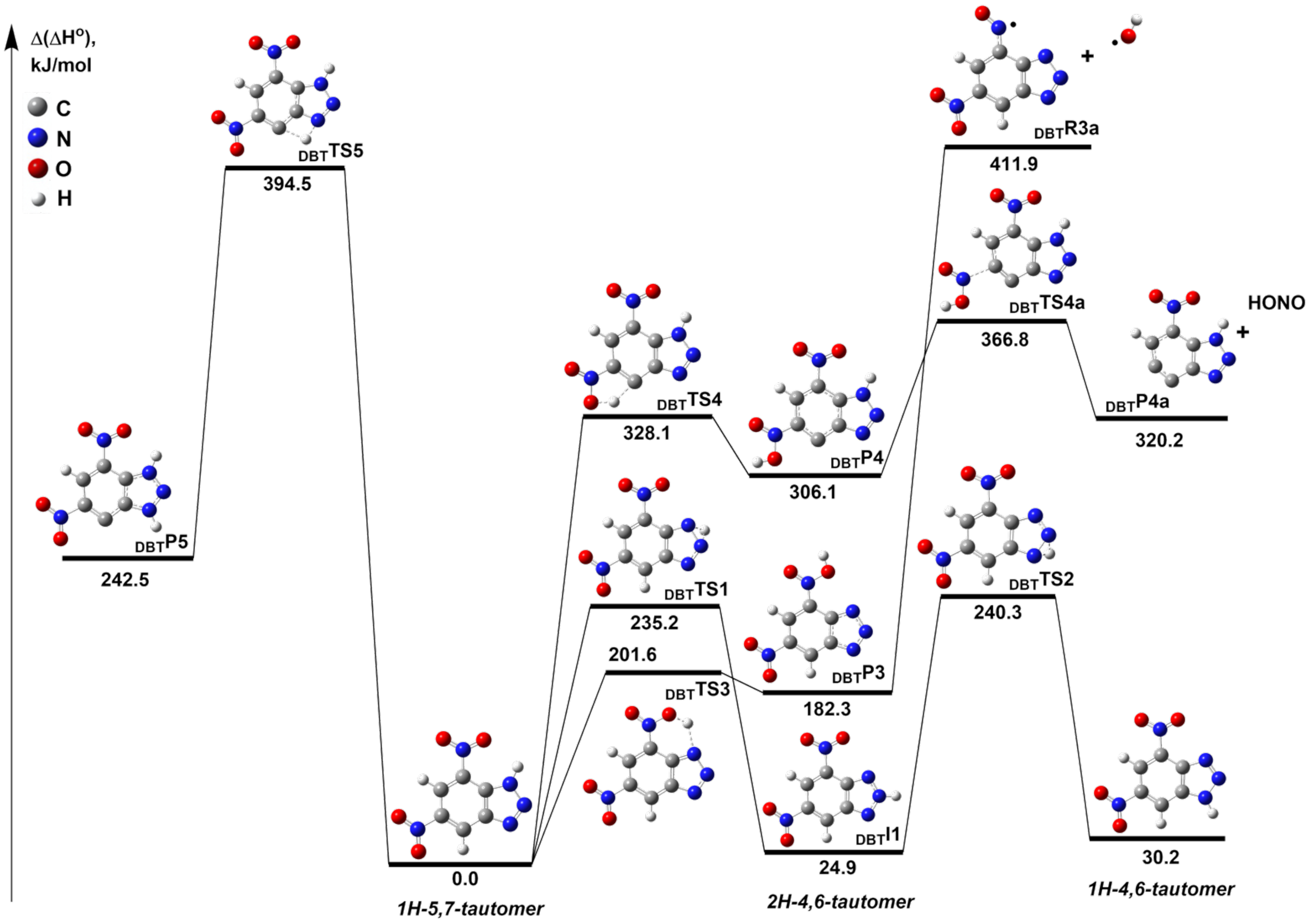

Figure S3. The relevant stationary points on the PES corresponding to the unimolecular tautomeric interconversions and aci-isomerization of **DBT**. The *1H-5,7-tautomer* of **DBT** was chosen as reference compound for the calculation of the relative enthalpies at 298 K ($\Delta(\Delta H°)$). The geometry optimizations were performed at the M06-2X/6-311++G(2df,p) level of theory, the thermal corrections were calculated at the same level of theory, and the single point energies were computed at the DLPNO-CCSD(T)/jun-VQZ level. All energy values are given in kJ mol^{-1}.

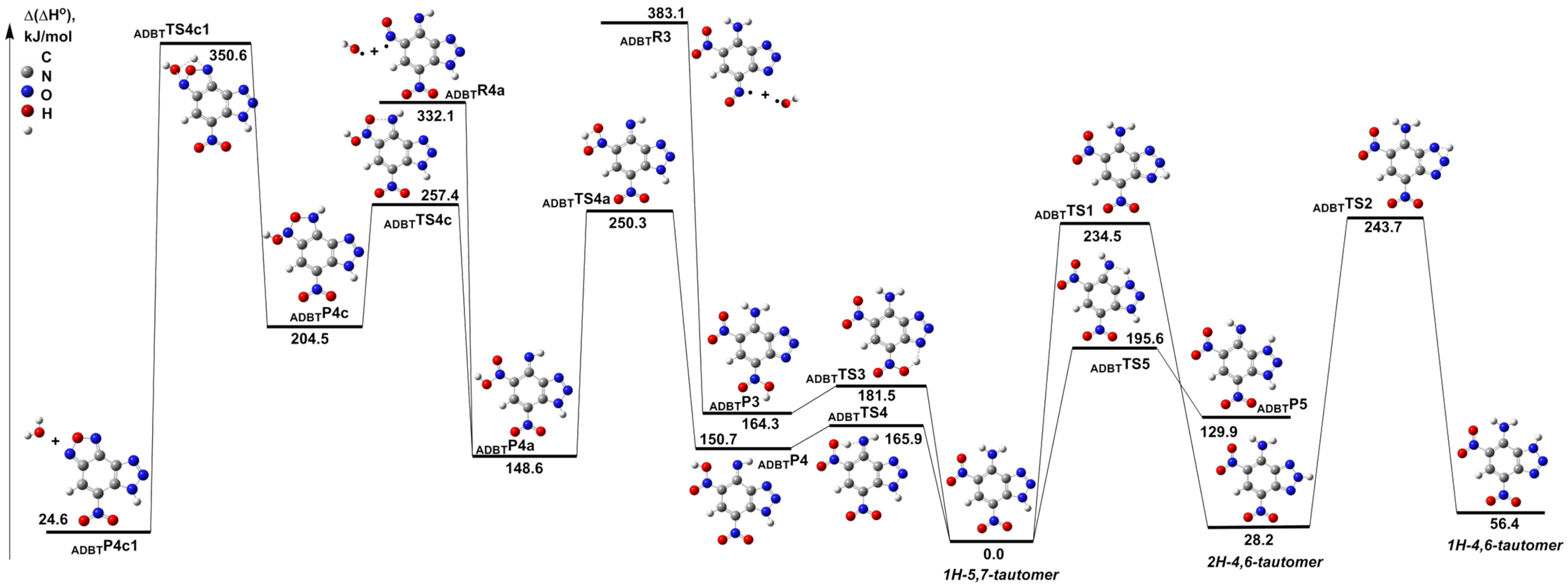

Figure S4. The relevant stationary points on the PES corresponding to the unimolecular tautomeric interconversions and aci-isomerization of **ADBT**. The *1H-5,7-tautomer* of **ADBT** was chosen as reference compound for the calculation of the relative enthalpies at 298 K (Δ(Δ*H*°)). The geometry optimizations were performed at the M06-2X/6-311++G(2df,p) level of theory, the thermal corrections were calculated at the same level of theory, and the single point energies were computed at the DLPNO-CCSD(T)/jun-VQZ level. All energy values are given in kJ mol^{-1}.

S4. Primary decomposition channels for *1H-4,6* and *2H-4,6-tautomers*

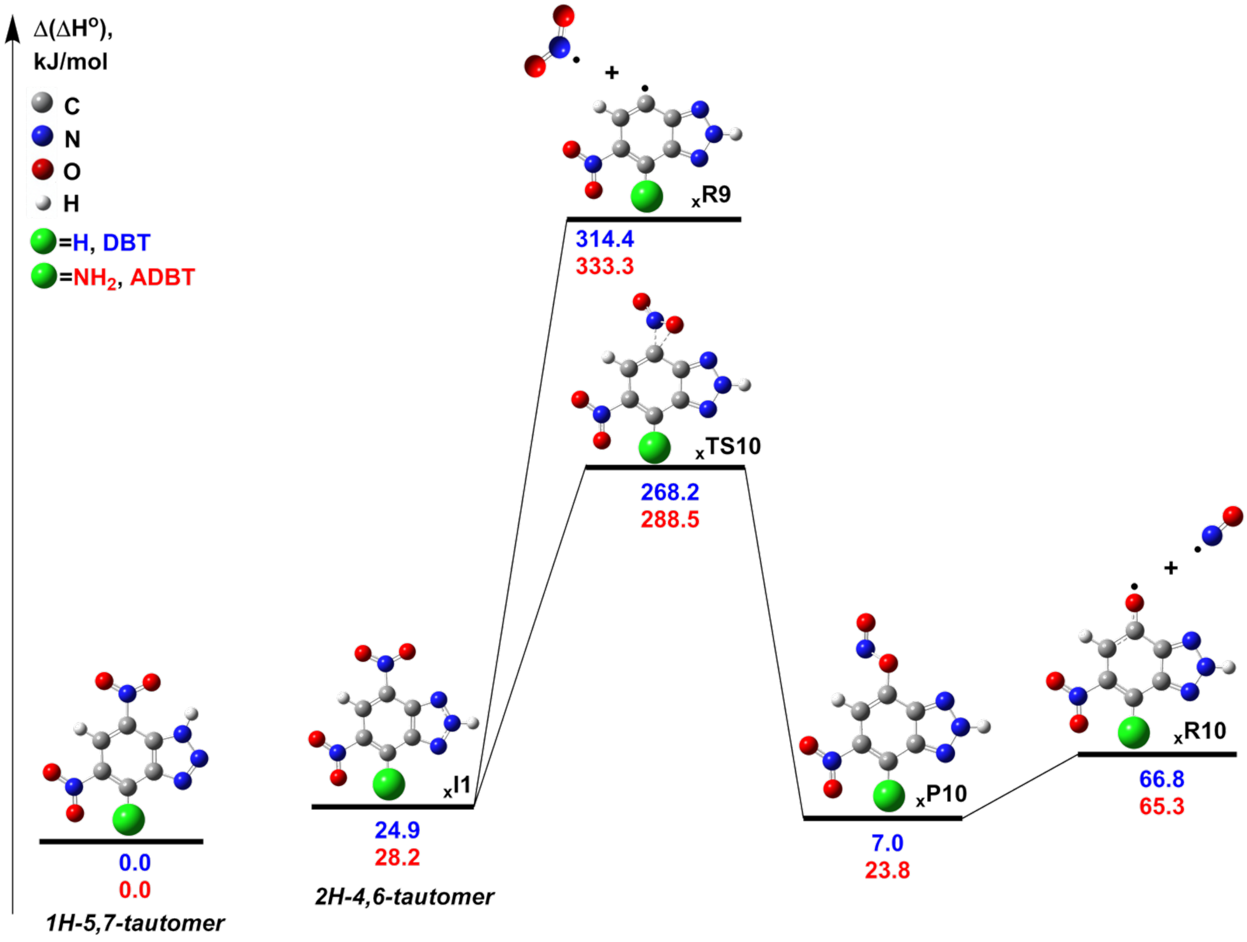

Figure S5. The relevant stationary points on the PES corresponding to the primary unimolecular decomposition channels of **DBT** and **ADBT** for *2H-46-tautomer*. The *1H-5,7-tautomer* was chosen as reference compound for the calculation of the relative enthalpies at 298 K ($\Delta(\Delta H°)$). The geometry optimizations were performed at the M06-2X/6-311++G(2df,p) level of theory, the thermal corrections were calculated at the same level of theory, and the single point energies were computed at the DLPNO-CCSD(T)/jun-VQZ level. All energy values are given in kJ mol^{-1}.

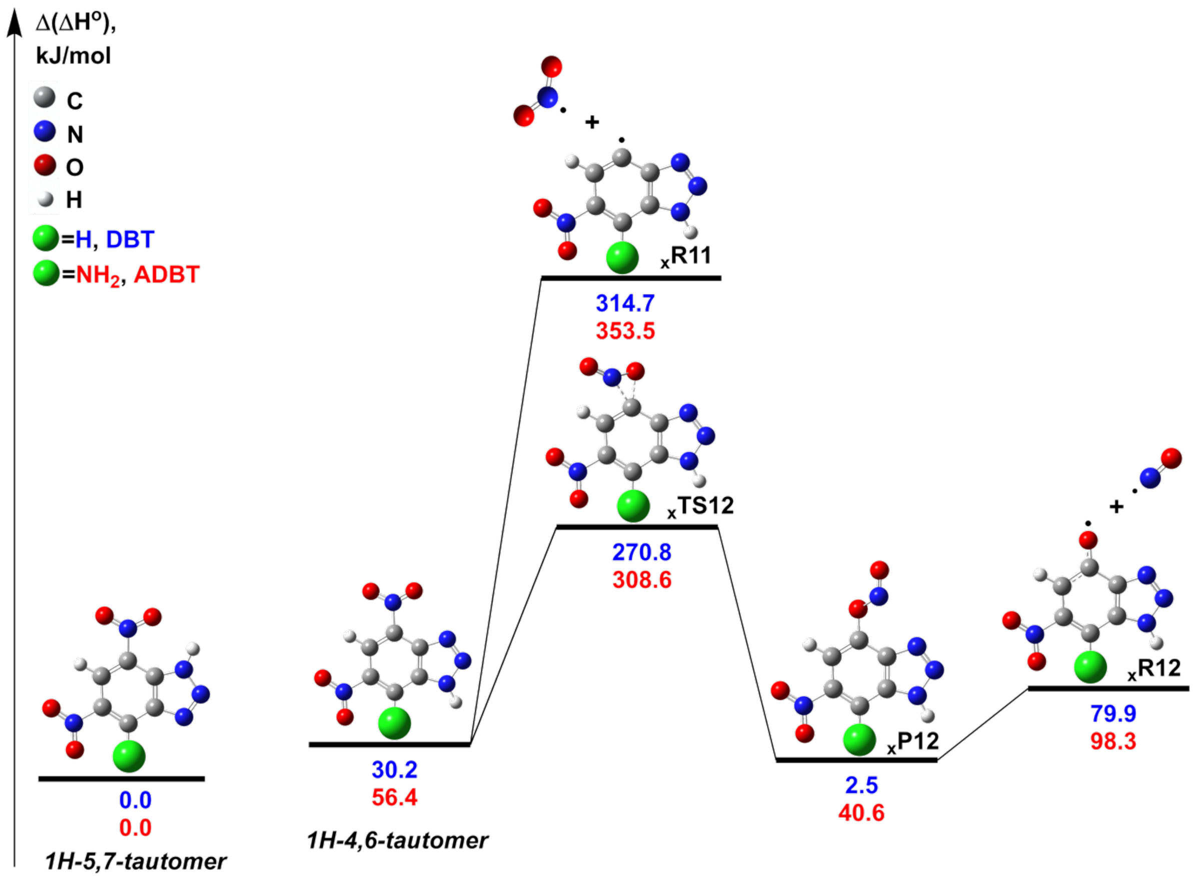

Figure S6. The relevant stationary points on the PES corresponding to the primary unimolecular decomposition channels of **DBT** and **ADBT** for *1H-46-tautomer*. The *1H-5,7-tautomer* was chosen as reference compound for the calculation of the relative enthalpies at 298 K (Δ(Δ*H*°)). The geometry optimizations were performed at the M06-2X/6-311++G(2df,p) level of theory, the thermal corrections were calculated at the same level of theory, and the single point energies were computed at the DLPNO-CCSD(T)/jun-VQZ level. All energy values are given in kJ mol

S5. The decomposition mechanism in solution based on PCM calculations

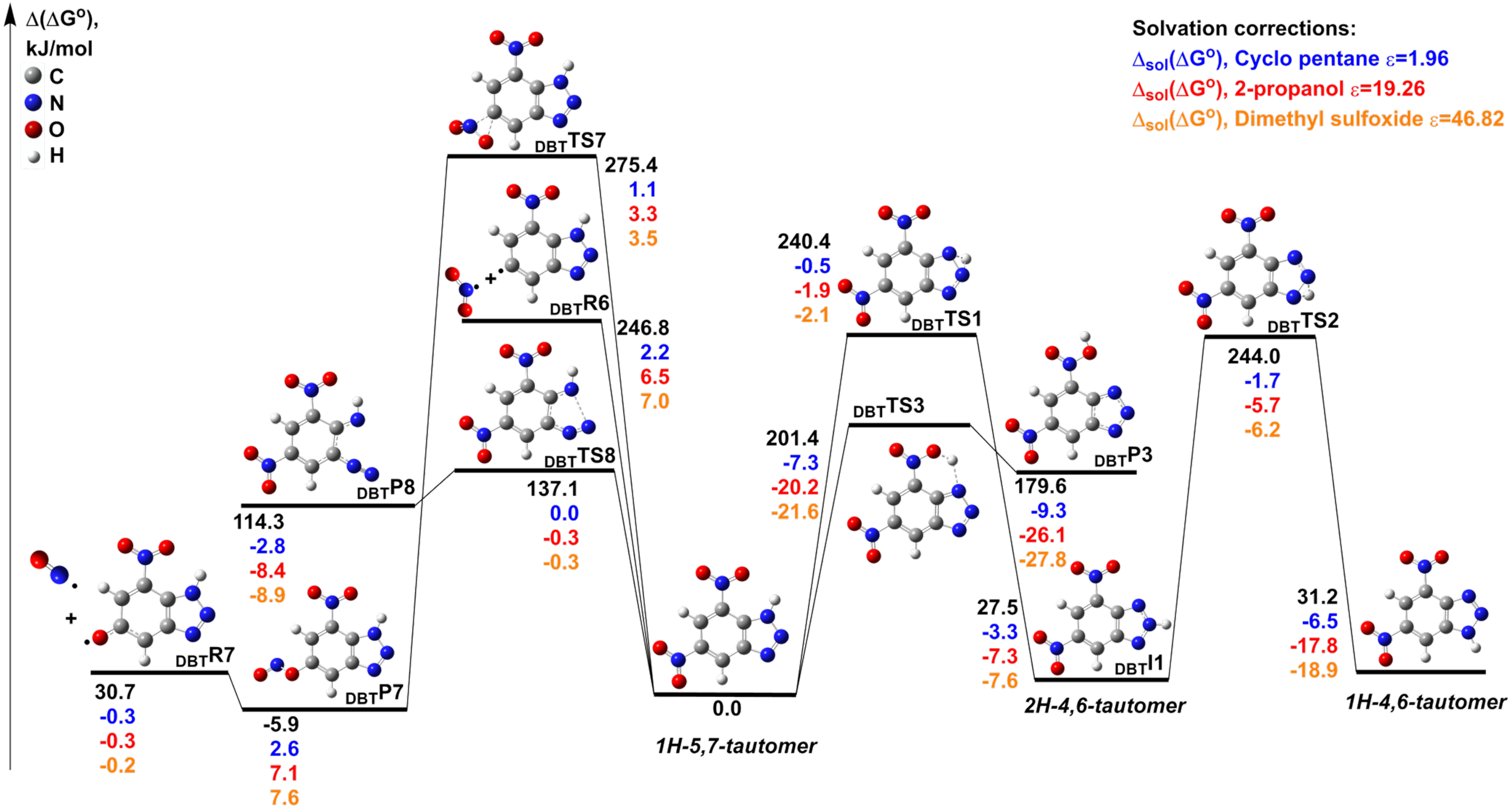

Figure S7. The stationary points on the PES for the most favourable decomposition channels of **DBT** in the three model solvents with the dielectric constants of ε = 1.96, 19.26 and 46.82. The relative free energies are calculated from the *1H-5,7-tautomer* of **DBT.** All values are calculated at the M06-2X/6-311++G(2df,p) level of theory and are given in kJ mol^{-1}.

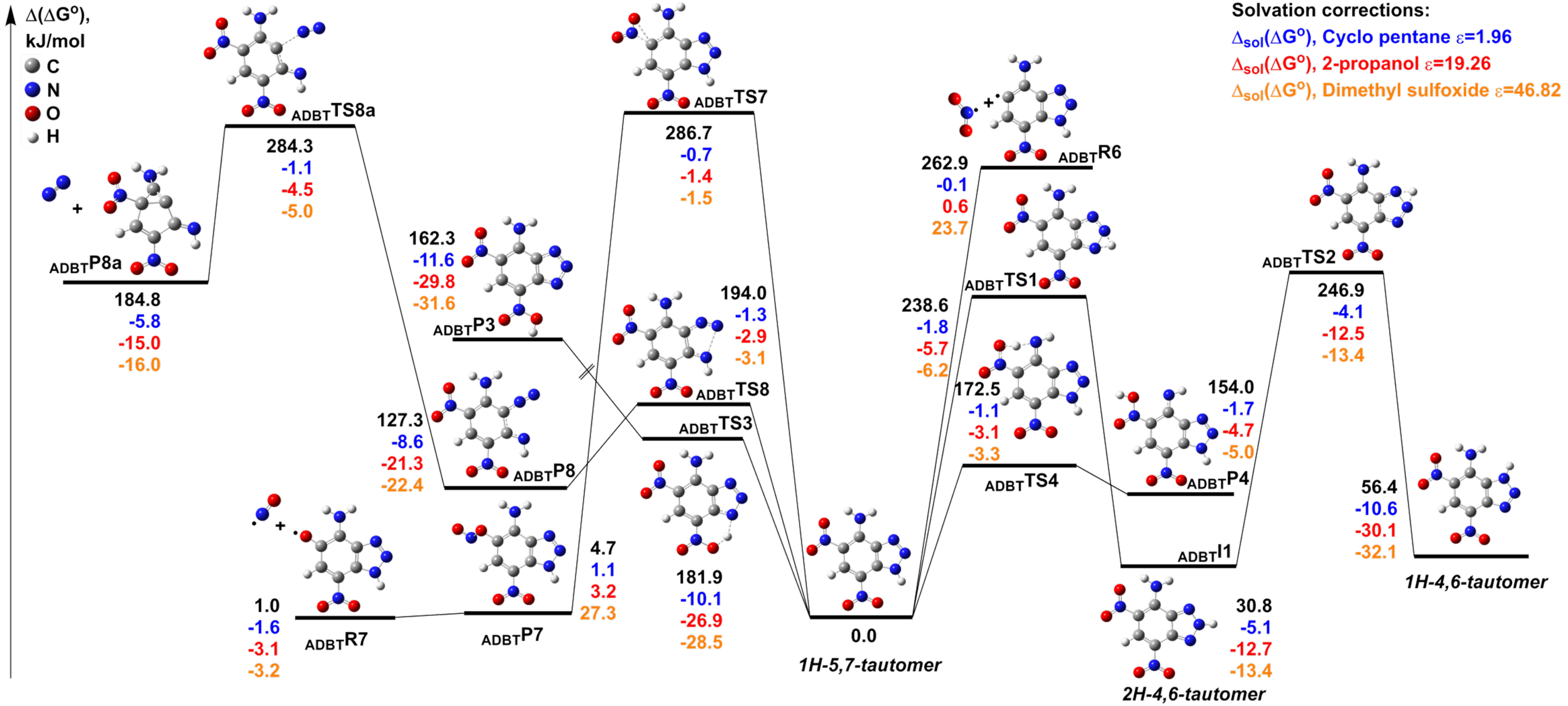

Figure S8. The stationary points on the PES for the most favourable decomposition channels of **ADBT** in the three model solvents with the dielectric constants of ε = 1.96, 19.26 and 46.82. The relative free energies are calculated from the *1H-5,7-tautomer* of **ADBT.** All values are calculated at the M06-2X/6-311++G(2df,p) level of theory and are given in kJ mol^{-1}.

S6. Kinetic parameters of the initial decomposition pathways of DBT and ADBT

Table S1. The activation enthalpies ($\Delta^{‡}H°$) at 298 K and Arrhenius parameters (the activation energies E_a and preexponential factor A) of gas phase decomposition channels: **xTS1, xTS2** – intramolecular hydrogen transfer; **xTS3, xTS4** – isomerization to aci-form; **xTS5** – hydrogen transfer from amino group to triazole ring; **xR6** - radical cleavage bond C-NO_2; $_{\mathbf{x}}$**TS7** - nitro-nitrite isomerization, **xTS8** – triazole ring opening.

Reaction	$\Delta^{‡}H°$, kJ mol^{-1}	E_a, kJ mol^{-1}	log(A/s^{-1})
**DBT→$_{\mathbf{DBT}}$P3 ($_{\mathbf{DBT}}$TS3)**	201.6	205.3	13.56
**DBT→$_{\mathbf{DBT}}$P4 ($_{\mathbf{DBT}}$TS4)**	328.1	332.5	13.45
**DBT→$_{\mathbf{DBT}}$P5 ($_{\mathbf{DBT}}$TS5)**	394.5	398.5	13.96
**DBT→•$_{\mathbf{DBT}}$R6+•NO_2 ($_{\mathbf{DBT}}$R6)**	299.2	297.6	18.10
**DBT→$_{\mathbf{DBT}}$P7 ($_{\mathbf{DBT}}$TS7)**	267.1	271.2	13.61
**DBT→$_{\mathbf{DBT}}$P8 ($_{\mathbf{DBT}}$TS8)**	143.0	147.3	14.03
**DBT→$_{\mathbf{DBT}}$P8a ($_{\mathbf{DBT}}$TS8a)**	281.4	289.4	18.11
**ADBT→$_{\mathbf{ADBT}}$P3 ($_{\mathbf{ADBT}}$TS3)**	181.5	184.5	13.54
**ADBT→$_{\mathbf{ADBT}}$P4 ($_{\mathbf{ADBT}}$TS4)**	165.9	168.6	13.40
**ADBT→$_{\mathbf{ADBT}}$P5 ($_{\mathbf{ADBT}}$TS5)**	195.6	197.7	12.96
**ADBT→•$_{\mathbf{ADBT}}$R6+•NO_2 ($_{\mathbf{ADBT}}$R6)**	318.7	319.8	19.11
**ADBT→$_{\mathbf{ADBT}}$P7 ($_{\mathbf{ADBT}}$TS7)**	276.2	280.3	13.88
**ADBT→$_{\mathbf{ADBT}}$P8 ($_{\mathbf{ADBT}}$TS8)**	149.2	153.1	14.28
**ADBT→$_{\mathbf{ADBT}}$P8a ($_{\mathbf{ADBT}}$TS8a)**	286.4	293.3	17.11

S7. Experimental thermochemical data of DBT

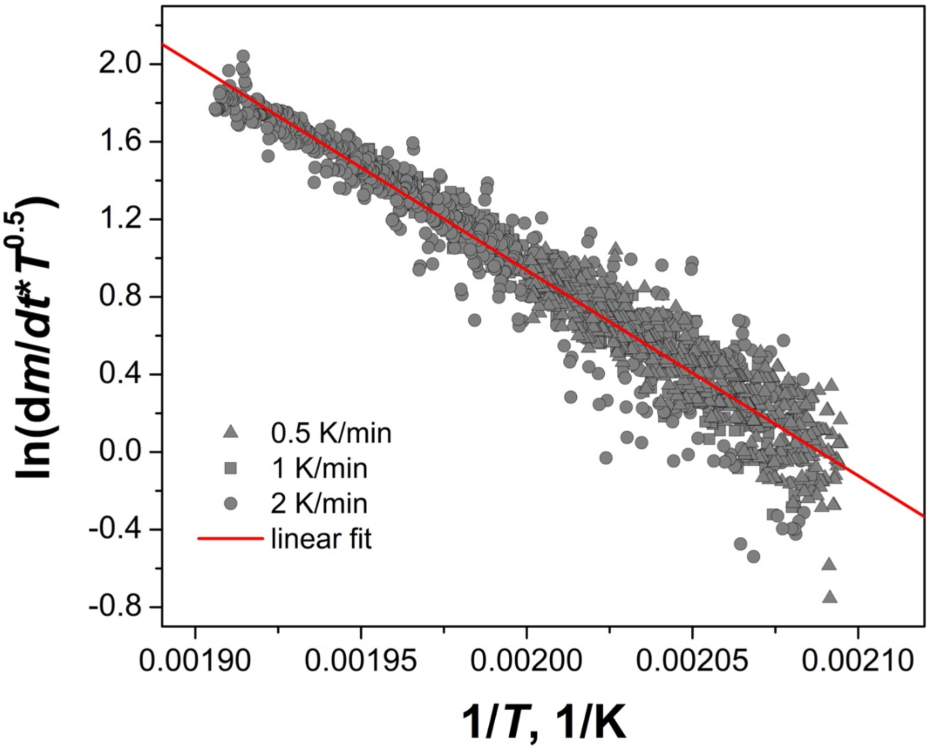

Figure S9. Vaporization data of **DBT**: transformed mass loss data below the melting point.

S8. Raw quantum chemical data: optimized geometries, electronic energies, and thermal corrections to thermodynamic potentials of all compounds under study

M06-2X/6-311++G(2df,p) geometries, zero-point vibrational energies (unscaled) and thermal corrections to thermodynamic potentials, M06-2X/6-311++G(2df,p) and DLPNO-CCSD(T)/ jun-cc-pVQZ electronic energies.

1H-5,7-dinitrobenzotriazole

Zero-point correction= 0.112992 (Hartree/Particle)
Thermal correction to Energy= 0.123765
Thermal correction to Enthalpy= 0.124709
Thermal correction to Gibbs Free Energy= 0.075026

Electronic energy:

M06-2X = -804.8386441
DLPNO-CCSD(T) = -803.875209948601

Center Number	Atomic Number	Atomic Type	Coordinates (Angstroms) X	Y	Z
1	7	0	1.067216	-2.746114	0.000004
2	7	0	2.404565	-1.071319	0.000012
3	6	0	0.684714	0.801729	0.000005
4	7	0	-2.973053	0.126031	-0.000016
5	6	0	-1.516889	-0.116456	-0.000008
6	8	0	-3.693823	-0.843700	-0.000020
7	8	0	-3.342031	1.276876	-0.000018
8	6	0	1.187114	-0.502315	0.000006
9	6	0	0.310248	-1.594485	0.000001
10	6	0	-0.671412	0.998791	-0.000003
11	7	0	2.295905	-2.417572	0.000010
12	7	0	1.604574	1.933610	0.000010
13	8	0	1.145305	3.046157	0.000016
14	8	0	2.790052	1.655825	0.000010
15	6	0	-1.069431	-1.418337	-0.000007
16	1	0	-1.081474	1.999298	-0.000004
17	1	0	-1.761295	-2.249279	-0.000011
18	1	0	3.306210	-0.617292	0.000016

1H-4,6-dinitrobenzotriazole

Zero-point correction= 0.112616 (Hartree/Particle)
Thermal correction to Energy= 0.123599
Thermal correction to Enthalpy= 0.124543
Thermal correction to Gibbs Free Energy= 0.073600

Electronic energy:

M06-2X = -804.8253483
DLPNO-CCSD(T) = -803.863535474869

Center Number	Atomic Number	Atomic Type	Coordinates (Angstroms) X	Y	Z

1	7	0	1.138566	-2.664411	-0.028610
2	7	0	2.505722	-1.015852	-0.035122
3	6	0	0.708346	0.798111	-0.004528
4	7	0	-2.953785	0.117682	0.020834
5	6	0	-1.494039	-0.121040	0.007766
6	8	0	-3.671222	-0.856032	0.025967
7	8	0	-3.326180	1.265451	0.025233
8	6	0	1.229943	-0.505176	-0.007688
9	6	0	0.326699	-1.577990	-0.006536
10	6	0	-0.646226	0.993402	-0.002061
11	7	0	2.423412	-2.291334	-0.047444
12	7	0	1.586964	1.981100	0.007333
13	8	0	1.070939	3.050934	-0.223671
14	8	0	2.750427	1.790624	0.253892
15	6	0	-1.056680	-1.421183	0.006283
16	1	0	-1.055532	1.993292	-0.006469
17	1	0	-1.759528	-2.242155	0.012499
18	1	0	0.908931	-3.645990	-0.035754

2H-4,6- dinitrobenzotriazole

Zero-point correction= 0.113704 (Hartree/Particle)
Thermal correction to Energy= 0.124519
Thermal correction to Enthalpy= 0.125463
Thermal correction to Gibbs Free Energy= 0.074885

Electronic energy:

M06-2X = -804.8280241
DLPNO-CCSD(T) = -803.866500531178

Center Number	Atomic Number	Atomic Type	Coordinates (Angstroms) X	Y	Z
1	7	0	1.065517	-2.731833	0.000092
2	7	0	2.489118	-0.982589	0.000105
3	6	0	0.698929	0.811096	0.000008
4	7	0	-2.957413	0.087953	-0.000103
5	6	0	-1.498841	-0.151087	-0.000048
6	8	0	-3.677209	-0.882902	-0.000101
7	8	0	-3.327994	1.237805	-0.000145
8	6	0	1.239307	-0.499098	0.000052
9	6	0	0.341400	-1.599376	0.000044
10	6	0	-0.649698	0.980006	-0.000041
11	7	0	2.285688	-2.278100	0.000130
12	7	0	1.562878	2.000508	0.000011
13	8	0	1.022285	3.082590	-0.000001
14	8	0	2.753283	1.802952	0.000025
15	6	0	-1.055005	-1.438330	-0.000007
16	1	0	-1.073264	1.974323	-0.000074
17	1	0	-1.739880	-2.274588	-0.000014
18	1	0	3.073180	-2.914136	0.000157

$_{DBT}$TS1

Zero-point correction= 0.106796 (Hartree/Particle)
Thermal correction to Energy= 0.117650
Thermal correction to Enthalpy= 0.118594
Thermal correction to Gibbs Free Energy= 0.068200

Electronic energy:

M06-2X = -804.7402642
DLPNO-CCSD(T) = -803.779504014775

Center Number	Atomic Number	Atomic Type	Coordinates (Angstroms) X	Y	Z
1	7	0	-1.045343	-2.744758	0.021336
2	7	0	-2.470144	-1.005845	-0.130856
3	6	0	-0.699172	0.804863	-0.012763
4	7	0	2.961115	0.110009	0.019206
5	6	0	1.501027	-0.138739	0.001727
6	8	0	3.684229	-0.856719	0.027074
7	8	0	3.321381	1.262560	0.024062
8	6	0	-1.224252	-0.507378	-0.027094
9	6	0	-0.333232	-1.600025	-0.005837
10	6	0	0.647685	0.988708	-0.005180
11	7	0	-2.290459	-2.402952	-0.104519
12	7	0	-1.583857	1.979031	0.011391
13	8	0	-1.078624	3.058979	-0.183791
14	8	0	-2.750589	1.766831	0.236328
15	6	0	1.064921	-1.428185	0.001480
16	1	0	1.064955	1.986056	-0.001369
17	1	0	1.752816	-2.261970	0.012558
18	1	0	-2.969999	-1.841165	0.729525

$_{DBT}$TS2

Zero-point correction= 0.106790 (Hartree/Particle)
Thermal correction to Energy= 0.117676
Thermal correction to Enthalpy= 0.118620
Thermal correction to Gibbs Free Energy= 0.067708

Electronic energy:

M06-2X = -804.7383849
DLPNO-CCSD(T) = -803.777613615216

Center Number	Atomic Number	Atomic Type	Coordinates (Angstroms) X	Y	Z
1	7	0	1.064655	-2.729145	-0.066922
2	7	0	2.493424	-0.992829	0.071639
3	6	0	0.705977	0.803175	0.001005
4	7	0	-2.956977	0.105308	-0.009829
5	6	0	-1.497889	-0.142073	-0.005332
6	8	0	-3.679243	-0.862842	-0.011432
7	8	0	-3.319163	1.257016	-0.010818
8	6	0	1.237052	-0.510458	0.008772

9	6	0	0.335544	-1.595754	-0.010301
10	6	0	-0.642092	0.982152	0.003202
11	7	0	2.396309	-2.288307	-0.023496
12	7	0	1.576308	1.991354	-0.009123
13	8	0	1.045297	3.064284	0.164109
14	8	0	2.750888	1.799772	-0.196327
15	6	0	-1.060380	-1.431537	-0.016384
16	1	0	-1.059119	1.979613	0.010797
17	1	0	-1.749027	-2.264572	-0.034776
18	1	0	1.940596	-3.018587	0.838075

DBTTS3

Zero-point correction= 0.109778 (Hartree/Particle)
Thermal correction to Energy= 0.120637
Thermal correction to Enthalpy= 0.121581
Thermal correction to Gibbs Free Energy= 0.071725
Electronic energy:
M06-2X = -804.7586368
DLPNO-CCSD(T) = -803.795282325643

Center Number	Atomic Number	Atomic Type	Coordinates (Angstroms) X	Y	Z
1	7	0	0.971391	2.821146	0.027476
2	6	0	0.734638	-0.760370	-0.020588
3	7	0	-2.964679	-0.209495	-0.010095
4	6	0	-1.516384	0.061985	-0.007711
5	8	0	-3.698502	0.751547	-0.017490
6	8	0	-3.318724	-1.365017	-0.003553
7	6	0	1.224668	0.588354	-0.020903
8	6	0	0.246908	1.641335	-0.020461
9	6	0	-0.666942	-0.996663	0.002760
10	7	0	2.196998	2.506070	0.067931
11	7	0	1.554436	-1.833323	0.004664
12	8	0	1.244533	-2.978829	0.172954
13	8	0	2.862922	-1.561450	-0.274600
14	1	0	-1.050303	-2.007870	0.006011
15	6	0	-1.088649	1.422524	-0.015359
16	7	0	2.411916	1.120225	0.039889
17	1	0	3.349176	-1.560455	0.567011
18	1	0	-1.816582	2.222970	-0.006995

DBTTS4

Zero-point correction= 0.109888 (Hartree/Particle)
Thermal correction to Energy= 0.120726
Thermal correction to Enthalpy= 0.121670
Thermal correction to Gibbs Free Energy= 0.072034
Electronic energy:
M06-2X = -804.7134037
DLPNO-CCSD(T) = -803.747213343426

Center Number	Atomic Number	Atomic Type	Coordinates (Angstroms) X	Y	Z
1	7	0	0.984565	-2.782616	0.031536
2	6	0	0.730097	0.794575	-0.011611
3	7	0	-2.821532	0.238419	0.017634
4	6	0	-1.479066	-0.095229	-0.028422
5	8	0	-3.711802	-0.756436	-0.263701
6	8	0	-3.277479	1.319608	0.219714
7	6	0	1.172429	-0.553813	0.004544
8	6	0	0.240843	-1.607565	0.006742
9	6	0	-0.599845	1.038189	-0.017535
10	7	0	2.217309	-2.503899	0.039390
11	7	0	1.692605	1.888433	-0.025658
12	8	0	1.277661	3.020033	-0.046777
13	8	0	2.866857	1.568518	-0.013290
14	1	0	-0.964416	2.056942	-0.032297
15	6	0	-1.151745	-1.476935	-0.013136
16	1	0	-4.082547	-1.049645	0.583756
17	7	0	2.362916	-1.140768	0.024087
18	1	0	3.277741	-0.713410	0.028558

$_{DBT}$TS5

Zero-point correction= 0.106643 (Hartree/Particle)
Thermal correction to Energy= 0.117583
Thermal correction to Enthalpy= 0.118527
Thermal correction to Gibbs Free Energy= 0.067661

Electronic energy:

M06-2X = -804.6833182
DLPNO-CCSD(T) = -803.718788807617

Center Number	Atomic Number	Atomic Type	Coordinates (Angstroms) X	Y	Z
1	7	0	-0.850330	-2.648080	0.000015
2	7	0	-2.386403	-1.223965	0.000117
3	6	0	-0.730061	0.825463	-0.000045
4	7	0	2.975357	0.170562	-0.000041
5	6	0	1.522217	-0.146938	-0.000079
6	8	0	3.738346	-0.760058	0.000063
7	8	0	3.291535	1.341942	-0.000118
8	6	0	-1.246745	-0.477668	-0.000051
9	6	0	-0.272220	-1.439074	-0.000122
10	6	0	0.643909	0.969787	-0.000070
11	7	0	-2.132312	-2.546925	0.000169
12	7	0	-1.643909	1.950690	0.000030
13	8	0	-1.198320	3.068469	0.000018
14	8	0	-2.828019	1.654348	0.000105
15	6	0	1.103054	-1.465015	-0.000096
16	1	0	1.058539	1.970118	-0.000064

17	1	0	0.401602	-3.020394	0.000072
18	1	0	-3.346230	-0.902640	0.000208

$_{DBT}$R6

Zero-point correction= 0.097280 (Hartree/Particle)
Thermal correction to Energy= 0.105453
Thermal correction to Enthalpy= 0.106397
Thermal correction to Gibbs Free Energy= 0.062834

Electronic energy:

M06-2X = -599.6524267
DLPNO-CCSD(T) = -598.894431839058

Center Number	Atomic Number	Atomic Type	Coordinates (Angstroms) X	Y	Z
1	7	0	-2.650756	-0.498668	-0.000004
2	7	0	-0.812364	-1.596693	-0.000003
3	6	0	0.815134	0.358664	0.000002
4	6	0	-0.391512	2.387518	0.000003
5	6	0	-0.412551	-0.307900	-0.000001
6	6	0	-1.615963	0.409067	-0.000002
7	6	0	0.831613	1.738377	0.000003
8	7	0	-2.154438	-1.673901	-0.000005
9	7	0	2.055340	-0.404917	0.000002
10	8	0	3.100613	0.196293	0.000005
11	8	0	1.941611	-1.617697	0.000001
12	6	0	-1.624977	1.811592	0.000000
13	1	0	1.778683	2.262642	0.000006
14	1	0	-2.552165	2.370755	-0.000001
15	1	0	-0.239248	-2.426817	-0.000003

$_{DBT}$TS7

Zero-point correction= 0.108966 (Hartree/Particle)
Thermal correction to Energy= 0.119949
Thermal correction to Enthalpy= 0.120893
Thermal correction to Gibbs Free Energy= 0.071120

Electronic energy:

M06-2X = -804.7298504
DLPNO-CCSD(T) = -803.769673092416

Center Number	Atomic Number	Atomic Type	Coordinates (Angstroms) X	Y	Z
1	7	0	-0.821673	2.771495	0.019644
2	7	0	-2.312252	1.237076	-0.055009
3	6	0	-0.763191	-0.789206	0.007719
4	7	0	3.068596	-0.440886	-0.264093
5	6	0	1.500728	-0.076462	0.115058
6	8	0	4.019256	0.012159	-0.802211
7	8	0	2.947066	-0.448625	1.019884

8	6	0	-1.148423	0.554994	-0.002041
9	6	0	-0.178495	1.557381	0.044617
10	6	0	0.562790	-1.119943	0.059633
11	7	0	-2.081018	2.557158	-0.038692
12	7	0	-1.778095	-1.838467	-0.049828
13	8	0	-1.416399	-2.987452	-0.045303
14	8	0	-2.932929	-1.459924	-0.098802
15	6	0	1.188100	1.260216	0.111962
16	1	0	0.870519	-2.155992	0.063315
17	1	0	1.946453	2.030215	0.143344
18	1	0	-3.250889	0.870008	-0.101026

$_{DBT}$TS8

Zero-point correction= 0.109211 (Hartree/Particle)
Thermal correction to Energy= 0.120388
Thermal correction to Enthalpy= 0.121332
Thermal correction to Gibbs Free Energy= 0.070577

Electronic energy:

M06-2X = -804.781973
DLPNO-CCSD(T) = -803.817371657293

Center Number	Atomic Number	Atomic Type	Coordinates (Angstroms) X	Y	Z
1	7	0	-0.978118	2.757331	-0.000001
2	7	0	-2.575349	0.884932	-0.000000
3	6	0	-0.731070	-0.811397	0.000000
4	7	0	2.936145	-0.136486	0.000000
5	6	0	1.500112	0.101293	0.000000
6	8	0	3.661521	0.836165	-0.000000
7	8	0	3.311820	-1.287203	0.000000
8	6	0	-1.361178	0.501545	-0.000000
9	6	0	-0.330125	1.561167	-0.000000
10	6	0	0.618844	-0.991047	0.000000
11	7	0	-2.033002	3.135091	-0.000001
12	7	0	-1.561324	-2.016652	0.000000
13	8	0	-1.004841	-3.089647	0.000001
14	8	0	-2.767904	-1.868555	0.000000
15	6	0	1.037118	1.392950	-0.000000
16	1	0	1.018950	-1.996345	0.000001
17	1	0	1.720725	2.231196	-0.000001
18	1	0	-3.265121	0.142491	-0.000000

$_{DBT}$TS8a

Zero-point correction= 0.102455 (Hartree/Particle)
Thermal correction to Energy= 0.116408
Thermal correction to Enthalpy= 0.117352
Thermal correction to Gibbs Free Energy= 0.057975

Electronic energy:

M06-2X = -804.7222373

DLPNO-CCSD(T) = -803.760666602201

Center Number	Atomic Number	Atomic Type	Coordinates (Angstroms) X	Y	Z
1	7	0	-2.450677	-2.720320	-0.140973
2	7	0	-2.699896	0.208996	0.547733
3	6	0	-0.307181	1.071277	0.053757
4	7	0	2.908122	-0.818825	0.074201
5	6	0	1.515819	-0.451806	-0.038520
6	8	0	3.183608	-1.992332	-0.066286
7	8	0	3.697994	0.071449	0.293569
8	6	0	-1.496741	0.082984	0.221376
9	6	0	-0.673368	-0.870560	-0.316850
10	6	0	1.058005	0.843950	0.096405
11	7	0	-3.395875	-3.248351	-0.066900
12	7	0	-0.718984	2.478059	-0.133192
13	8	0	0.166488	3.280505	-0.314409
14	8	0	-1.897014	2.751673	-0.085141
15	6	0	0.532606	-1.429661	-0.238652
16	1	0	1.736753	1.682790	0.157453
17	1	0	-2.938932	1.148959	0.846619
18	1	0	0.719896	-2.496135	-0.257128

4-amino-5,7-dinitrobenzotriazole (1H-5,7-ADBT)
Zero-point correction= 0.130835 (Hartree/Particle)
Thermal correction to Energy= 0.142762
Thermal correction to Enthalpy= 0.143707
Thermal correction to Gibbs Free Energy= 0.091927
Electronic energy:
M06-2X = -860.2155825
DLPNO-CCSD(T) = -859.184719021999

Center Number	Atomic Number	Atomic Type	Coordinates (Angstroms) X	Y	Z
1	7	0	0.698786	2.738015	0.000049
2	6	0	0.995098	-0.816941	-0.000059
3	7	0	-2.707292	-0.884257	0.000005
4	6	0	-1.369934	-0.321613	-0.000010
5	8	0	-3.649921	-0.105895	0.000361
6	8	0	-2.827886	-2.086050	-0.000274
7	6	0	1.239879	0.568751	-0.000001
8	6	0	0.189383	1.469570	0.000039
9	6	0	-0.306267	-1.234116	-0.000064
10	7	0	1.974552	2.659088	0.000095
11	7	0	2.093774	-1.751005	-0.000073
12	8	0	1.854949	-2.933780	-0.000087
13	8	0	3.212688	-1.260242	-0.000059
14	1	0	-0.529469	-2.292606	-0.000100
15	6	0	-1.175647	1.079607	0.000018

16	7	0	-2.106160	2.029796	-0.000110
17	1	0	-1.802581	2.991688	0.000156
18	1	0	-3.082887	1.784654	0.000302
19	7	0	2.327056	1.359432	0.000094
20	1	0	3.296195	1.074965	0.000157

4-amino-4,6-1H-dinitrobenzotriazole (1H-4,6-ADBT)

Zero-point correction= 0.129197 (Hartree/Particle)
Thermal correction to Energy= 0.141878
Thermal correction to Enthalpy= 0.142823
Thermal correction to Gibbs Free Energy= 0.088320

Electronic energy:

M06-2X = -860.19051
DLPNO-CCSD(T) = -859.162362847786

Center Number	Atomic Number	Atomic Type	Coordinates (Angstroms) X	Y	Z
1	7	0	0.753351	2.699965	-0.008364
2	7	0	2.417671	1.360791	-0.005645
3	6	0	1.029509	-0.788998	-0.000600
4	7	0	-2.670933	-0.916589	-0.000350
5	6	0	-1.341280	-0.324613	-0.003472
6	8	0	-3.633680	-0.161391	0.003824
7	8	0	-2.763702	-2.118733	-0.001308
8	6	0	1.273238	0.604024	0.001142
9	6	0	0.183015	1.466510	-0.001326
10	6	0	-0.259806	-1.223175	-0.005998
11	7	0	2.085656	2.596801	-0.013301
12	7	0	2.110774	-1.772534	0.004099
13	8	0	1.800201	-2.943971	-0.025614
14	8	0	3.238611	-1.344125	0.038505
15	1	0	-0.466148	-2.283899	-0.008989
16	1	0	0.345448	3.620629	-0.036580
17	6	0	-1.177202	1.069229	0.003547
18	7	0	-2.154121	1.993363	-0.000788
19	1	0	-1.937434	2.968310	0.097864
20	1	0	-3.114952	1.690294	0.035130

4-amino-4,6-2H-dinitrobenzotriazole (2H-4,6-ADBT)

Zero-point correction= 0.131286 (Hartree/Particle)
Thermal correction to Energy= 0.143277
Thermal correction to Enthalpy= 0.144221
Thermal correction to Gibbs Free Energy= 0.091853

Electronic energy:

M06-2X = -860.2037942
DLPNO-CCSD(T) = -859.17449463877

Center Number	Atomic Number	Atomic Type	Coordinates (Angstroms) X	Y	Z

1	7	0	-0.641495	2.736078	0.000008
2	6	0	-1.026609	-0.804596	-0.000007
3	7	0	2.680596	-0.897142	0.000002
4	6	0	1.354348	-0.316154	0.000000
5	8	0	3.636579	-0.132312	0.000007
6	8	0	2.784967	-2.100532	-0.000001
7	6	0	-1.285062	0.596360	0.000001
8	6	0	-0.186922	1.479836	0.000005
9	6	0	0.265272	-1.218611	-0.000008
10	7	0	-1.934284	2.562578	0.000005
11	7	0	-2.098461	-1.788748	-0.000012
12	8	0	-1.791922	-2.961555	-0.000075
13	8	0	-3.229485	-1.358741	0.000049
14	1	0	0.480316	-2.278382	-0.000016
15	6	0	1.185773	1.073228	0.000007
16	7	0	2.124431	2.017483	0.000015
17	1	0	1.835368	2.982680	0.000020
18	1	0	3.098153	1.758449	0.000017
19	7	0	-2.400495	1.334248	0.000002
20	1	0	-2.567795	3.350507	0.000008

ADBTTS1

Zero-point correction= 0.124574 (Hartree/Particle)
Thermal correction to Energy= 0.136550
Thermal correction to Enthalpy= 0.137494
Thermal correction to Gibbs Free Energy= 0.085247

Electronic energy:

M06-2X = -860.1180409
DLPNO-CCSD(T) = -859.089181902165

Center Number	Atomic Number	Atomic Type	Coordinates (Angstroms) X	Y	Z
1	7	0	-0.629857	2.740788	0.034749
2	6	0	-1.024231	-0.801945	-0.013703
3	7	0	2.683529	-0.905561	0.008713
4	6	0	1.355096	-0.322598	-0.003128
5	8	0	3.636504	-0.139317	0.017634
6	8	0	2.785274	-2.108373	0.008951
7	6	0	-1.273889	0.596204	-0.027859
8	6	0	-0.187461	1.478493	-0.002935
9	6	0	0.267202	-1.222753	-0.006678
10	7	0	-1.925447	2.683987	-0.085518
11	7	0	-2.106699	-1.774210	0.009635
12	8	0	-1.816203	-2.946043	-0.083396
13	8	0	-3.227082	-1.332043	0.129007
14	1	0	0.479395	-2.283493	-0.000616
15	6	0	1.188200	1.067434	-0.001434
16	7	0	2.121105	2.014059	0.006786
17	1	0	1.823570	2.977844	0.006851

18	1	0	3.096791	1.762185	0.013847
19	7	0	-2.385108	1.346809	-0.120728
20	1	0	-2.679852	2.239562	0.761303

ADBTTS2

Zero-point correction= 0.124399 (Hartree/Particle)
Thermal correction to Energy= 0.136443
Thermal correction to Enthalpy= 0.137387
Thermal correction to Gibbs Free Energy= 0.084666
Electronic energy:
M06-2X = -860.1142967
DLPNO-CCSD(T) = -859.085587151331

Center Number	Atomic Number	Atomic Type	Coordinates (Angstroms) X	Y	Z
1	7	0	0.640496	2.737212	-0.071511
2	6	0	1.033254	-0.792847	-0.003167
3	7	0	-2.675637	-0.913523	0.006868
4	6	0	-1.349937	-0.322829	0.004435
5	8	0	-3.633742	-0.153184	0.003046
6	8	0	-2.770387	-2.116459	0.012951
7	6	0	1.281117	0.607374	0.002575
8	6	0	0.183053	1.477565	-0.008484
9	6	0	-0.257787	-1.216067	0.003737
10	7	0	2.046295	2.598331	-0.046682
11	7	0	2.109709	-1.777351	-0.008560
12	8	0	1.798049	-2.948191	0.023374
13	8	0	3.238549	-1.349437	-0.045879
14	1	0	-0.465305	-2.277653	0.008087
15	6	0	-1.188520	1.067730	-0.007214
16	7	0	-2.133642	2.005989	-0.017724
17	1	0	-1.856024	2.973166	-0.069163
18	1	0	-3.105353	1.738836	-0.037006
19	7	0	2.404256	1.348842	0.046118
20	1	0	1.419513	3.181764	0.839282

ADBTTS3

Zero-point correction= 0.128095 (Hartree/Particle)
Thermal correction to Energy= 0.140023
Thermal correction to Enthalpy= 0.140968
Thermal correction to Gibbs Free Energy= 0.088854
Electronic energy:
M06-2X = -860.143236
DLPNO-CCSD(T) = -859.112862154223

Center Number	Atomic Number	Atomic Type	Coordinates (Angstroms) X	Y	Z
1	7	0	0.629464	2.785128	0.034295

2	6	0	1.031727	-0.751925	-0.018004
3	7	0	-2.698705	-0.938760	-0.009300
4	6	0	-1.359548	-0.344555	-0.005022
5	8	0	-3.650465	-0.177587	-0.010229
6	8	0	-2.791826	-2.140106	-0.010351
7	6	0	1.271115	0.662855	-0.024666
8	6	0	0.160141	1.514100	-0.020105
9	6	0	-0.311601	-1.210522	0.006316
10	7	0	1.922806	2.698803	0.069833
11	7	0	2.002024	-1.680419	0.003767
12	8	0	1.885842	-2.866881	0.176538
13	8	0	3.250756	-1.198391	-0.277828
14	1	0	-0.509826	-2.274080	0.009737
15	6	0	-1.175312	1.100219	-0.015770
16	7	0	-2.143012	1.997597	-0.010556
17	1	0	-1.865131	2.970513	-0.011983
18	1	0	-3.112162	1.721633	0.000538
19	7	0	2.359257	1.416687	0.037173
20	1	0	3.730685	-1.128621	0.563693

ADBTTS4

Zero-point correction= 0.128977 (Hartree/Particle)
Thermal correction to Energy= 0.140795
Thermal correction to Enthalpy= 0.141739
Thermal correction to Gibbs Free Energy= 0.089873

Electronic energy:

M06-2X = -860.1478404
DLPNO-CCSD(T) = -859.119552984553

Center Number	Atomic Number	Atomic Type	Coordinates (Angstroms) X	Y	Z
1	7	0	0.837100	2.740213	0.045783
2	6	0	0.955386	-0.836067	-0.026700
3	7	0	-2.626384	-0.794648	0.055505
4	6	0	-1.399671	-0.269425	-0.011070
5	8	0	-3.622921	0.059307	0.440546
6	8	0	-2.934444	-1.957248	-0.100980
7	6	0	1.256811	0.564588	0.027040
8	6	0	0.263436	1.512377	0.009956
9	6	0	-0.330686	-1.228911	-0.054355
10	7	0	2.116313	2.595642	0.093797
11	7	0	2.030424	-1.813123	-0.020100
12	8	0	1.751940	-2.984980	-0.069563
13	8	0	3.160553	-1.359290	0.035860
14	1	0	-0.573496	-2.282995	-0.066298
15	6	0	-1.159692	1.200831	-0.107486
16	7	0	-2.072540	2.047744	-0.360924
17	1	0	-1.658616	2.979723	-0.427980
18	1	0	-4.177831	0.209491	-0.340769
19	7	0	2.388813	1.285123	0.079735

20	1	0	3.339338	0.944459	0.107262

$_{ADBT}$TS4a

Zero-point correction= 0.125264 (Hartree/Particle)
Thermal correction to Energy= 0.137136
Thermal correction to Enthalpy= 0.138080
Thermal correction to Gibbs Free Energy= 0.086022
Electronic energy:
M06-2X = -860.1107007
DLPNO-CCSD(T) = -859.083745940422

Center Number	Atomic Number	Atomic Type	Coordinates (Angstroms) X	Y	Z
1	7	0	1.024388	2.713417	0.000099
2	6	0	0.907980	-0.865974	-0.000033
3	7	0	-2.645426	-0.560638	-0.000017
4	6	0	-1.401717	-0.147750	-0.000004
5	8	0	-3.731462	0.126772	0.000011
6	8	0	-3.045318	-1.803979	-0.000061
7	6	0	1.299393	0.515059	0.000017
8	6	0	0.370713	1.526421	0.000056
9	6	0	-0.396103	-1.182920	-0.000043
10	7	0	2.292364	2.484633	0.000088
11	7	0	1.922532	-1.911250	-0.000074
12	8	0	1.567903	-3.062468	-0.000116
13	8	0	3.078535	-1.527941	-0.000060
14	1	0	-0.705955	-2.219320	-0.000081
15	6	0	-1.074432	1.307723	0.000050
16	7	0	-1.967726	2.207142	0.000084
17	1	0	-1.522577	3.127839	0.000117
18	1	0	-4.095200	-1.092624	-0.000033
19	7	0	2.477670	1.159681	0.000039
20	1	0	3.404862	0.758785	0.000023

$_{ADBT}$TS4c

Zero-point correction= 0.127404 (Hartree/Particle)
Thermal correction to Energy= 0.138896
Thermal correction to Enthalpy= 0.139840
Thermal correction to Gibbs Free Energy= 0.089399
Electronic energy:
M06-2X = -860.1027607
DLPNO-CCSD(T) = -859.082832888053

Center Number	Atomic Number	Atomic Type	Coordinates (Angstroms) X	Y	Z
1	7	0	-0.368436	2.745259	0.143470
2	6	0	-1.099383	-0.751952	-0.132638
3	7	0	2.571325	-1.006801	-0.144448

4	6	0	1.265089	-0.554473	-0.345664
5	8	0	3.426641	-0.151700	-0.449022
6	8	0	2.702421	-1.587140	1.129967
7	6	0	-1.152242	0.651619	0.002942
8	6	0	-0.005293	1.437076	-0.015896
9	6	0	0.125384	-1.350006	-0.273960
10	7	0	-1.640526	2.796677	0.267218
11	7	0	-2.308318	-1.535983	-0.077297
12	8	0	-2.228238	-2.737058	-0.185281
13	8	0	-3.349047	-0.916454	0.077651
14	1	0	0.197657	-2.428686	-0.314866
15	6	0	1.278374	0.873801	-0.248362
16	7	0	2.442762	1.470148	-0.339330
17	1	0	2.584157	2.267843	0.282872
18	1	0	3.551390	-2.048768	1.092796
19	7	0	-2.138124	1.550566	0.177647
20	1	0	-3.129761	1.372966	0.243325

ADBTTS4c1

Zero-point correction= 0.124170 (Hartree/Particle)
Thermal correction to Energy= 0.135317
Thermal correction to Enthalpy= 0.136261
Thermal correction to Gibbs Free Energy= 0.086549

Electronic energy:

M06-2X = -860.0660324
DLPNO-CCSD(T) = -859.043732527095

Center Number	Atomic Number	Atomic Type	Coordinates (Angstroms) X	Y	Z
1	7	0	-0.088856	2.707255	0.023450
2	6	0	-1.184361	-0.718299	-0.084185
3	7	0	2.458421	-1.304650	-0.325112
4	6	0	1.158151	-0.791014	-0.318560
5	8	0	3.202644	-0.320497	-0.804469
6	8	0	2.822466	-0.866628	1.435642
7	6	0	-1.083101	0.687380	-0.021443
8	6	0	0.145178	1.360296	-0.088755
9	6	0	-0.046897	-1.477750	-0.204489
10	7	0	-1.345118	2.882861	0.144351
11	7	0	-2.487027	-1.351923	0.000375
12	8	0	-2.545880	-2.555143	-0.059137
13	8	0	-3.443911	-0.607680	0.129797
14	1	0	-0.103558	-2.557709	-0.211040
15	6	0	1.301040	0.597367	-0.264383
16	7	0	2.644914	0.903816	-0.255438
17	1	0	2.857836	0.354910	0.849496
18	1	0	3.709398	-1.238623	1.506652
19	7	0	-1.969881	1.687741	0.116535
20	1	0	-2.973471	1.617429	0.202009

ADBTTS5

Zero-point correction= 0.126623 (Hartree/Particle)
Thermal correction to Energy= 0.138121
Thermal correction to Enthalpy= 0.139065
Thermal correction to Gibbs Free Energy= 0.087975

Electronic energy:

M06-2X = -860.1478404
DLPNO-CCSD(T) = -859.119552984553

Center Number	Atomic Number	Atomic Type	Coordinates (Angstroms) X	Y	Z
1	7	0	-0.534907	2.609970	0.000014
2	6	0	-1.014573	-0.852659	-0.000010
3	7	0	2.741211	-0.902200	-0.000002
4	6	0	1.404671	-0.363064	-0.000005
5	8	0	3.647986	-0.086391	0.000008
6	8	0	2.892856	-2.104043	-0.000010
7	6	0	-1.268662	0.540474	-0.000004
8	6	0	-0.171301	1.335088	-0.000002
9	6	0	0.314021	-1.245835	-0.000011
10	7	0	-1.825297	2.717598	0.000022
11	7	0	-2.110839	-1.764443	-0.000013
12	8	0	-1.899559	-2.953958	-0.000019
13	8	0	-3.224124	-1.246915	-0.000008
14	1	0	0.530925	-2.307920	-0.000015
15	6	0	1.222737	1.054762	0.000003
16	7	0	1.864941	2.184491	0.000019
17	1	0	0.586931	3.076252	0.000025
18	1	0	2.876281	2.145900	0.000025
19	7	0	-2.275278	1.458379	0.000010
20	1	0	-3.271589	1.277078	0.000012

ADBTR6

Zero-point correction= 0.113955 (Hartree/Particle)
Thermal correction to Energy= 0.124007
Thermal correction to Enthalpy= 0.124951
Thermal correction to Gibbs Free Energy= 0.077296

Electronic energy:

M06-2X = -655.0207972
DLPNO-CCSD(T) = -654.196075711191

Center Number	Atomic Number	Atomic Type	Coordinates (Angstroms) X	Y	Z
1	7	0	-2.014262	1.465631	0.000006
2	6	0	0.986751	-0.477255	-0.000005
3	6	0	-0.834397	-2.002743	0.000006
4	6	0	0.036574	0.551517	-0.000010
5	6	0	-1.329189	0.278637	-0.000011

6	6	0	0.518625	-1.785168	0.000007
7	7	0	-1.163567	2.419624	0.000013
8	7	0	2.388704	-0.173947	-0.000000
9	8	0	3.184979	-1.085725	0.000007
10	8	0	2.690211	1.012019	-0.000004
11	1	0	1.234035	-2.598937	0.000018
12	6	0	-1.832816	-1.043579	-0.000009
13	7	0	-3.157924	-1.282803	-0.000085
14	1	0	-3.800853	-0.508520	0.000231
15	1	0	-3.513712	-2.221745	0.000307
16	7	0	0.077001	1.900159	0.000001
17	1	0	0.896056	2.489741	0.000005

ADBTTS7

Zero-point correction= 0.126486 (Hartree/Particle)
Thermal correction to Energy= 0.138777
Thermal correction to Enthalpy= 0.139721
Thermal correction to Gibbs Free Energy= 0.087279
Electronic energy:
M06-2X = -860.0977966
DLPNO-CCSD(T) = -859.071265133719

Center Number	Atomic Number	Atomic Type	Coordinates (Angstroms) X	Y	Z
1	7	0	-0.546141	2.812872	-0.080652
2	6	0	-1.083709	-0.697191	0.091943
3	7	0	2.592623	-1.026995	0.019452
4	6	0	1.306965	-0.369123	0.034407
5	8	0	3.593215	-0.324112	-0.033175
6	8	0	2.624607	-2.236402	0.061010
7	6	0	-1.228745	0.697330	0.033354
8	6	0	-0.119250	1.516622	-0.023396
9	6	0	0.171339	-1.219070	0.112486
10	7	0	-1.824545	2.826517	-0.055041
11	7	0	-2.340792	-1.645486	-0.256897
12	8	0	-2.511248	-2.682914	-0.812019
13	8	0	-2.285908	-1.559016	1.033491
14	1	0	0.330507	-2.287560	0.146894
15	6	0	1.220116	1.028204	-0.030777
16	7	0	2.214240	1.919443	-0.104633
17	1	0	1.975393	2.897024	-0.152275
18	1	0	3.171495	1.609593	-0.109929
19	7	0	-2.265096	1.555211	0.012443
20	1	0	-3.255054	1.368934	0.070048

ADBTTS8

Zero-point correction= 0.126435 (Hartree/Particle)
Thermal correction to Energy= 0.138928
Thermal correction to Enthalpy= 0.139872

Thermal correction to Gibbs Free Energy= 0.086641

Electronic energy:

M06-2X = -860.1541012

DLPNO-CCSD(T) = -859.124071448177

Center Number	Atomic Number	Atomic Type	Coordinates (Angstroms) X	Y	Z
1	7	0	-0.497294	2.764360	0.000003
2	7	0	-2.475421	1.320169	-0.000000
3	6	0	-1.077624	-0.752102	-0.000002
4	7	0	2.620632	-1.017352	0.000001
5	6	0	1.331558	-0.382528	0.000001
6	8	0	3.618370	-0.301580	0.000003
7	8	0	2.670987	-2.225660	-0.000001
8	6	0	-1.381721	0.668052	-0.000000
9	6	0	-0.143345	1.468050	0.000002
10	6	0	0.202455	-1.212283	-0.000001
11	7	0	-1.435277	3.386893	0.000003
12	7	0	-2.147281	-1.736357	-0.000004
13	8	0	-1.847333	-2.909236	-0.000006
14	8	0	-3.293481	-1.324839	-0.000005
15	6	0	1.193482	1.022182	0.000002
16	1	0	0.364779	-2.282178	-0.000003
17	1	0	-3.311796	0.746765	-0.000002
18	7	0	2.198785	1.899494	0.000005
19	1	0	2.015383	2.888355	0.000006
20	1	0	3.145452	1.548893	0.000005

$_{ADBT}$TS8a

Zero-point correction= 0.121667 (Hartree/Particle)

Thermal correction to Energy= 0.136224

Thermal correction to Enthalpy= 0.137168

Thermal correction to Gibbs Free Energy= 0.077952

Electronic energy:

M06-2X = -860.0933267

DLPNO-CCSD(T) = -859.069083902699

Center Number	Atomic Number	Atomic Type	Coordinates (Angstroms) X	Y	Z
1	7	0	0.506104	3.330632	-0.145013
2	7	0	2.420412	1.265444	0.257946
3	6	0	1.105734	-0.874888	0.102054
4	7	0	-2.589917	-1.122813	0.050477
5	6	0	-1.289850	-0.504932	0.005765
6	8	0	-3.573877	-0.395868	-0.021292
7	8	0	-2.647556	-2.327794	0.132480
8	6	0	1.353936	0.574363	0.295672
9	6	0	0.065012	1.183252	0.569309
10	6	0	-0.158702	-1.348097	0.000665

11	7	0	1.025563	4.283316	-0.115093
12	7	0	2.199607	-1.804617	-0.116740
13	8	0	1.939692	-2.984563	-0.181184
14	8	0	3.322843	-1.343977	-0.209020
15	6	0	-1.160909	0.912964	-0.006628
16	1	0	-0.321579	-2.411151	-0.116979
17	1	0	3.218004	0.668256	0.052160
18	7	0	-2.097761	1.799456	-0.338748
19	1	0	-1.921311	2.779654	-0.199941
20	1	0	-3.043321	1.464952	-0.453919

$_{DBT}$D1

Zero-point correction= 0.227031 (Hartree/Particle)
Thermal correction to Energy= 0.250730
Thermal correction to Enthalpy= 0.251674
Thermal correction to Gibbs Free Energy= 0.167548

Electronic energy:

M06-2X = -1609.691157
DLPNO-CCSD(T) = -1607.76782145157

Center Number	Atomic Number	Atomic Type	Coordinates (Angstroms) X	Y	Z
1	7	0	2.131455	1.827805	-0.000000
2	7	0	1.662536	-0.270439	0.000004
3	6	0	4.031783	-1.191699	0.000002
4	7	0	7.018894	1.027017	-0.000006
5	6	0	5.603829	0.604592	-0.000003
6	8	0	7.239681	2.214809	-0.000008
7	8	0	7.855362	0.155057	-0.000006
8	6	0	3.004701	-0.241775	0.000002
9	6	0	3.313555	1.125684	-0.000001
10	6	0	5.334185	-0.770556	-0.000001
11	7	0	1.176260	0.984777	0.000002
12	7	0	3.707615	-2.615247	0.000005
13	8	0	4.614618	-3.407364	0.000004
14	8	0	2.523294	-2.889570	0.000007
15	6	0	4.631467	1.576061	-0.000003
16	1	0	6.142852	-1.488448	-0.000001
17	1	0	4.886922	2.626683	-0.000005
18	1	0	1.014274	-1.053348	0.000005
19	7	0	-2.131452	-1.827803	0.000001
20	7	0	-1.662536	0.270442	0.000003
21	6	0	-4.031784	1.191700	0.000001
22	7	0	-7.018893	-1.027020	-0.000004
23	6	0	-5.603828	-0.604594	-0.000002
24	8	0	-7.239678	-2.214812	-0.000004
25	8	0	-7.855361	-0.155062	-0.000004
26	6	0	-3.004702	0.241777	0.000002
27	6	0	-3.313553	-1.125683	0.000000
28	6	0	-5.334186	0.770555	-0.000001

29	7	0	-1.176258	-0.984773	0.000003
30	7	0	-3.707618	2.615248	0.000003
31	8	0	-4.614622	3.407364	0.000002
32	8	0	-2.523297	2.889572	0.000004
33	6	0	-4.631465	-1.576061	-0.000001
34	1	0	-6.142854	1.488446	-0.000001
35	1	0	-4.886919	-2.626684	-0.000003
36	1	0	-1.014275	1.053352	0.000004

$_{DBT}TS_{D12}$

Zero-point correction= 0.218565 (Hartree/Particle)
Thermal correction to Energy= 0.241861
Thermal correction to Enthalpy= 0.242805
Thermal correction to Gibbs Free Energy= 0.161880

Electronic energy:

M06-2X = -1609.6536945
DLPNO-CCSD(T) = -1607.72802881143

Center Number	Atomic Number	Atomic Type	Coordinates (Angstroms) X	Y	Z
1	7	0	-1.916412	1.898147	0.000026
2	7	0	-1.362833	-0.237829	0.000067
3	6	0	-3.709585	-1.186636	0.000022
4	7	0	-6.760056	0.947735	-0.000069
5	6	0	-5.331474	0.571555	-0.000035
6	8	0	-7.020110	2.127510	-0.000116
7	8	0	-7.566958	0.048460	-0.000049
8	6	0	-2.700540	-0.206933	0.000031
9	6	0	-3.059582	1.157387	0.000008
10	6	0	-5.017961	-0.800807	-0.000010
11	7	0	-0.966377	1.040260	0.000059
12	7	0	-3.360794	-2.608092	0.000044
13	8	0	-4.260522	-3.411041	0.000052
14	8	0	-2.176261	-2.869021	0.000051
15	6	0	-4.395732	1.570679	-0.000027
16	1	0	-5.807158	-1.539904	-0.000016
17	1	0	-4.683723	2.612807	-0.000047
18	1	0	-0.297529	-1.014169	0.000052
19	7	0	1.916412	-1.898150	-0.000002
20	7	0	1.362831	0.237831	0.000046
21	6	0	3.709583	1.186632	0.000020
22	7	0	6.760056	-0.947732	-0.000069
23	6	0	5.331476	-0.571554	-0.000039
24	8	0	7.020114	-2.127508	-0.000118
25	8	0	7.566961	-0.048459	-0.000042
26	6	0	2.700537	0.206928	0.000020
27	6	0	3.059584	-1.157391	-0.000010
28	6	0	5.017962	0.800806	-0.000009
29	7	0	0.966370	-1.040263	0.000030
30	7	0	3.360799	2.608089	0.000049

31	8	0	4.260530	3.411035	0.000049
32	8	0	2.176268	2.869031	0.000070
33	6	0	4.395733	-1.570680	-0.000041
34	1	0	5.807157	1.539903	-0.000008
35	1	0	4.683726	-2.612807	-0.000066
36	1	0	0.297382	1.014236	0.000060

$_{DBT}$D2

Zero-point correction= 0.228564 (Hartree/Particle)
Thermal correction to Energy= 0.252017
Thermal correction to Enthalpy= 0.252961
Thermal correction to Gibbs Free Energy= 0.169974

Electronic energy:

M06-2X = -1609.6837106
DLPNO-CCSD(T) = -1607.76093929361

Center Number	Atomic Number	Atomic Type	Coordinates (Angstroms) X	Y	Z
1	7	0	2.137577	2.115628	0.000000
2	7	0	1.373288	0.006881	0.000003
3	6	0	3.624266	-1.120303	0.000002
4	7	0	6.861824	0.718706	-0.000004
5	6	0	5.402537	0.483528	-0.000002
6	8	0	7.236909	1.867090	-0.000006
7	8	0	7.577024	-0.255255	-0.000005
8	6	0	2.707401	-0.045918	0.000002
9	6	0	3.198174	1.282591	0.000000
10	6	0	4.959598	-0.858803	0.000000
11	7	0	1.130665	1.298154	0.000002
12	7	0	3.156617	-2.509799	0.000004
13	8	0	3.986612	-3.383899	0.000005
14	8	0	1.954548	-2.684300	0.000004
15	6	0	4.573039	1.567628	-0.000002
16	1	0	5.679112	-1.665489	0.000000
17	1	0	4.963386	2.575807	-0.000003
18	1	0	0.155505	1.607898	0.000002
19	7	0	-2.137577	-2.115628	0.000003
20	7	0	-1.373288	-0.006881	0.000002
21	6	0	-3.624266	1.120303	-0.000000
22	7	0	-6.861824	-0.718706	-0.000003
23	6	0	-5.402537	-0.483528	-0.000001
24	8	0	-7.236909	-1.867090	-0.000002
25	8	0	-7.577024	0.255255	-0.000005
26	6	0	-2.707401	0.045918	0.000001
27	6	0	-3.198174	-1.282591	0.000001
28	6	0	-4.959598	0.858803	-0.000001
29	7	0	-1.130665	-1.298154	0.000003
30	7	0	-3.156617	2.509799	-0.000000
31	8	0	-3.986612	3.383899	-0.000003
32	8	0	-1.954548	2.684300	0.000002

33	6	0	-4.573039	-1.567628	0.000000
34	1	0	-5.679112	1.665489	-0.000003
35	1	0	-4.963386	-2.575807	0.000000
36	1	0	-0.155505	-1.607898	0.000003

$_{DBT}$D3

Zero-point correction= 0.228108 (Hartree/Particle)
Thermal correction to Energy= 0.251923
Thermal correction to Enthalpy= 0.252867
Thermal correction to Gibbs Free Energy= 0.167030

Electronic energy:

M06-2X = -1609.6706018
DLPNO-CCSD(T) = -1607.7503221995

Center Number	Atomic Number	Atomic Type	Coordinates (Angstroms) X	Y	Z
1	7	0	1.523680	0.291715	-0.000150
2	7	0	2.306073	-1.814072	-0.000180
3	6	0	4.761339	-1.182165	-0.000406
4	7	0	5.996624	2.334790	-0.000576
5	6	0	5.049935	1.197764	-0.000473
6	8	0	5.523236	3.446515	-0.000558
7	8	0	7.174584	2.070027	-0.000671
8	6	0	3.359562	-0.980377	-0.000289
9	6	0	2.867782	0.349889	-0.000270
10	6	0	5.593341	-0.107208	-0.000496
11	7	0	1.291947	-0.994894	-0.000105
12	7	0	5.345246	-2.532494	-0.000436
13	8	0	6.551317	-2.616299	-0.000492
14	8	0	4.568419	-3.455381	-0.000402
15	6	0	3.716117	1.470758	-0.000364
16	1	0	6.665048	-0.247671	-0.000585
17	1	0	3.349624	2.487725	-0.000351
18	1	0	0.324176	-1.319208	-0.000013
19	7	0	-1.523680	-0.291719	0.000139
20	7	0	-2.306071	1.814069	0.000170
21	6	0	-4.761338	1.182165	0.000407
22	7	0	-5.996626	-2.334788	0.000587
23	6	0	-5.049936	-1.197764	0.000478
24	8	0	-5.523240	-3.446514	0.000568
25	8	0	-7.174587	-2.070024	0.000687
26	6	0	-3.359561	0.980375	0.000282
27	6	0	-2.867782	-0.349891	0.000263
28	6	0	-5.593341	0.107209	0.000503
29	7	0	-1.291946	0.994891	0.000093
30	7	0	-5.345243	2.532496	0.000435
31	8	0	-6.551314	2.616301	0.000537
32	8	0	-4.568416	3.455381	0.000355
33	6	0	-3.716119	-1.470759	0.000362
34	1	0	-6.665048	0.247674	0.000599

35	1	0	-3.349627	-2.487726	0.000348
36	1	0	-0.324175	1.319203	-0.000002

$_{DBT}TS_{D34}$

Zero-point correction= 0.217652 (Hartree/Particle)
Thermal correction to Energy= 0.241460
Thermal correction to Enthalpy= 0.242404
Thermal correction to Gibbs Free Energy= 0.157015

Electronic energy:

M06-2X = -1609.6380645
DLPNO-CCSD(T) = -1607.71410224572

Center Number	Atomic Number	Atomic Type	Coordinates (Angstroms) X	Y	Z
1	7	0	-1.364042	0.357333	-0.000155
2	7	0	-2.036346	-1.741533	-0.000123
3	6	0	-4.523740	-1.209761	0.000418
4	7	0	-5.919494	2.245744	0.000912
5	6	0	-4.924981	1.148743	0.000637
6	8	0	-5.492171	3.375873	0.000824
7	8	0	-7.084854	1.931770	0.001206
8	6	0	-3.137165	-0.945519	0.000139
9	6	0	-2.711402	0.400736	0.000127
10	6	0	-5.408946	-0.173579	0.000678
11	7	0	-1.037995	-0.929245	-0.000285
12	7	0	-5.047873	-2.586440	0.000460
13	8	0	-6.249389	-2.720458	0.000866
14	8	0	-4.233165	-3.475099	0.000069
15	6	0	-3.601508	1.482598	0.000365
16	1	0	-6.472708	-0.364008	0.000907
17	1	0	-3.283687	2.515677	0.000349
18	1	0	0.248590	-1.007271	-0.000523
19	7	0	1.363906	-0.357426	-0.000631
20	7	0	2.036405	1.741582	-0.000321
21	6	0	4.523840	1.209780	-0.000276
22	7	0	5.919357	-2.245737	-0.000556
23	6	0	4.925042	-1.148692	-0.000502
24	8	0	5.491993	-3.375901	-0.000755
25	8	0	7.084822	-1.932026	-0.000397
26	6	0	3.137243	0.945703	-0.000422
27	6	0	2.711337	-0.400516	-0.000595
28	6	0	5.409072	0.173554	-0.000283
29	7	0	1.037769	0.929264	-0.000461
30	7	0	5.048172	2.586350	-0.000166
31	8	0	6.249747	2.720299	0.001095
32	8	0	4.233686	3.475230	-0.001396
33	6	0	3.601446	-1.482317	-0.000667
34	1	0	6.472823	0.363957	-0.000135
35	1	0	3.283564	-2.515373	-0.000827
36	1	0	-0.254384	1.005886	-0.000299

$_{DBT}$D4

Zero-point correction= 0.226343 (Hartree/Particle)
Thermal correction to Energy= 0.250389
Thermal correction to Enthalpy= 0.251334
Thermal correction to Gibbs Free Energy= 0.164458

Electronic energy:

M06-2X = -1609.6697558
DLPNO-CCSD(T) = -1607.74896614552

Center Number	Atomic Number	Atomic Type	Coordinates (Angstroms) X	Y	Z
1	7	0	-1.619960	-0.370456	0.013239
2	7	0	-2.232237	1.690584	0.010016
3	6	0	-4.738073	1.207540	-0.001936
4	7	0	-6.207353	-2.216245	-0.020548
5	6	0	-5.194704	-1.136444	-0.011151
6	8	0	-5.798342	-3.353531	-0.016590
7	8	0	-7.367209	-1.883700	-0.031618
8	6	0	-3.363008	0.915417	0.012145
9	6	0	-2.971969	-0.432680	0.011589
10	6	0	-5.650446	0.189810	-0.017751
11	7	0	-1.222868	0.902157	0.011069
12	7	0	-5.232443	2.595592	0.009190
13	8	0	-6.415469	2.760018	-0.179971
14	8	0	-4.417224	3.459550	0.212288
15	6	0	-3.873381	-1.496983	0.003722
16	1	0	-6.709449	0.404005	-0.033811
17	1	0	-3.579561	-2.537117	0.007162
18	1	0	-0.895927	-1.085161	0.013272
19	7	0	1.619960	0.370456	0.013233
20	7	0	2.232237	-1.690584	0.010040
21	6	0	4.738073	-1.207540	-0.001920
22	7	0	6.207353	2.216245	-0.020584
23	6	0	5.194704	1.136444	-0.011170
24	8	0	5.798342	3.353531	-0.016642
25	8	0	7.367209	1.883700	-0.031649
26	6	0	3.363008	-0.915417	0.012157
27	6	0	2.971969	0.432680	0.011581
28	6	0	5.650446	-0.189810	-0.017750
29	7	0	1.222868	-0.902156	0.011082
30	7	0	5.232443	-2.595592	0.009226
31	8	0	6.415469	-2.760021	-0.179934
32	8	0	4.417224	-3.459547	0.212338
33	6	0	3.873381	1.496983	0.003698
34	1	0	6.709449	-0.404006	-0.033807
35	1	0	3.579561	2.537117	0.007123
36	1	0	0.895927	1.085161	0.013256
